# Alterações Longitudinais nos Níveis de Atividade Física e Parâmetros de Risco Cardiovascular em Pacientes com Doença Arterial Periférica Sintomática

**DOI:** 10.36660/abc.20210386

**Published:** 2022-05-24

**Authors:** Francielly Monteiro, Marilia de Almeida Correia, Breno Quintella Farah, Diego Giuliano Destro Christofaro, Paulo Mesquita Longano de Oliveira, Raphael Mendes Ritti-Dias, Gabriel Grizzo Cucato

**Affiliations:** 1 Hospital Israelita Albert Einstein Instituto de Educação e Pesquisa São Paulo SP Brasil Hospital Israelita Albert Einstein - Instituto de Educação e Pesquisa, São Paulo, SP – Brasil; 2 Universidade Nove de Julho Programa de Pós-graduação em Medicina São Paulo SP Brasil Universidade Nove de Julho - Programa de Pós-graduação em Medicina, São Paulo, SP – Brasil; 3 Universidade Federal Rural de Pernambuco Recife PE Brasil Universidade Federal Rural de Pernambuco, Recife, PE – Brasil; 4 Universidade Estadual Paulista Júlio de Mesquita Filho Presidente Prudente SP Brasil Universidade Estadual Paulista Júlio de Mesquita Filho, Presidente Prudente, SP – Brasil; 5 Northumbria University Newcastle upon Tyne Reino Unido Northumbria University, Newcastle upon Tyne – Reino Unido

**Keywords:** Doença Arterial Periférica, Sistema Cardiovascular, Pressão Arterial, Exercício Físico

## Abstract

**Fundamento:**

Estudos transversais anteriores demonstraram que a atividade física está associada a menor risco cardiovascular em pacientes com doença arterial periférica (DAP). No entanto, não é possível estabelecer causalidade e estudos com desenho longitudinal são necessários.

**Objetivo:**

Analisar as alterações nos parâmetros de risco cardiovascular e níveis de atividade física após 2 anos de acompanhamento em pacientes com DAP sintomática.

**Métodos:**

O presente estudo teve início em 2015. Na primeira fase, foram incluídos 268 pacientes. Na segunda fase, após 2 anos (mediana = 26 meses), foram reavaliados 72 pacientes. Parâmetros de risco cardiovascular, como pressão arterial, modulação autonômica cardíaca e rigidez arterial, e níveis de atividade física foram medidos na linha de base e após 2 anos de acompanhamento. A associação entre as alterações delta (valores após 2 anos – valores da linha de base) na atividade física e nos parâmetros cardiovasculares foi analisada por meio de regressão linear múltipla. O nível de significância foi estabelecido em p < 0,05 com DAP.

**Resultados:**

Pacientes reduziram seus níveis totais de atividade física em comparação com a linha de base (linha de base = 2.257,6 ± 774,5 versus acompanhamento = 2.041 ± 676,2 min/semana, p = 0,001). Após o acompanhamento, o índice tornozelo-braquial (0,62 ± 0,20 versus 0,54 ± 0,20, p = 0,003) e o desvio padrão de todos os intervalos RR (43,4 ± 27,0 versus 25,1 ± 13,4 ms, p < 0,001) foram menores, enquanto a velocidade da onda de pulso carotídeo-femoral foi maior (9,0 ± 3,0 versus 10,7 ± 3,4 m/s, p = 0,002) em relação aos valores basais. Não observamos associação entre os valores delta dos níveis de atividade física e os parâmetros de risco cardiovascular.

**Conclusão:**

Pacientes com DAP tiveram níveis reduzidos de atividade física e comprometimento em relação aos parâmetros de risco cardiovascular após 2 anos de acompanhamento.

## Introdução

A claudicação intermitente é o principal sintoma da doença arterial periférica (DAP), e é caracterizada por dor, cãibras ou sensação de queimação que acomete os membros inferiores durante a atividade física, principalmente durante a caminhada.^1^ Os pacientes com DAP e sintomas de claudicação intermitente apresentam mobilidade limitada, controle deficiente dos parâmetros cardiovasculares,^2,3^ e qualidade de vida comprometida.^4,5^

A atividade física tem sido recomendada para melhorar a capacidade funcional e a função cardiovascular desses pacientes.^6-8^ De fato, pacientes com DAP sintomática e níveis mais elevados de atividade física apresentam maior capacidade funcional e menor risco de mortalidade cardiovascular em comparação com pacientes sedentários.^9,10^ No entanto, devido ao desenho transversal desses estudos, não é possível estabelecer causalidade, e estudos de desenho longitudinal são necessários. Além disso, não se sabe se ocorrem alterações nesses parâmetros durante o acompanhamento em pacientes com DAP.

Portanto, o presente estudo visou analisar as alterações longitudinais na atividade física e nos parâmetros de risco cardiovascular após 2 anos de acompanhamento em pacientes com DAP. Também analisamos se as alterações nos níveis de atividade física estão associadas a alterações nos parâmetros de risco cardiovascular após 2 anos de acompanhamento. Nossa hipótese foi de que as alterações nos níveis de atividade física estariam associadas a melhores parâmetros de risco cardiovascular.

## Métodos

Trata-se de um estudo longitudinal que teve início em 2015, composto por 2 fases. Na primeira fase do estudo, 268 pacientes foram incluídos e submetidos à medidas de atividade física (acelerometria), capacidade funcional e parâmetros de risco cardiovascular (pressão arterial clínica, pressão arterial central, modulação autonômica cardíaca e rigidez arterial). Após 2 anos, todos os pacientes incluídos na primeira fase foram convidados para a fase 2.

### Recrutamento, triagem e dimensionamento de amostras

Os pacientes foram recrutados em hospitais de São Paulo, Brasil. Os critérios de inclusão foram: idade > 45 anos de ambos os sexos, índice tornozelo-braquial (ITB) < 0,90 em um ou ambos os membros e presença de sintomas de claudicação intermitente. O presente estudo foi aprovado pelo Comitê de Ética Institucional. Antes da coleta dos dados, os pacientes foram informados sobre os procedimentos envolvidos no estudo e assinaram o Termo de Consentimento Livre e Esclarecido.

Antes e após o acompanhamento de 2 anos, os pacientes foram avaliados em 2 visitas com intervalo de pelo menos 7 dias. Na primeira consulta, foram obtidos dados clínicos, sociodemográficos e de capacidade funcional, e todos os pacientes receberam um monitor de atividade física acelerômetro triaxial GT3X+ (Actigraph, Pensacola, FL, EUA). Durante a segunda visita, foram obtidas medidas de parâmetros de risco cardiovascular, como pressão arterial clínica, pressão arterial central, modulação autonômica cardíaca e rigidez arterial. Esta sessão começou entre as 13:00 e as 14:00 horas, e os pacientes receberam as seguintes orientações: fazer uma refeição leve, não realizar exercício pelo menos 24 horas antes do dia da avaliação, não ingerir bebidas alcoólicas ou cafeinadas, não fumar 12 horas antes da sessão e manter uma rotina normal de comer e tomar a sua medicação.

### Nível de atividade física

Os níveis de atividade física foram obtidos por meio de um acelerômetro triaxial GT3X+ (Actigraph, Pensacola, FL, EUA). Todos os pacientes receberam instruções para usar o acelerômetro durante 7 dias consecutivos, retirando-o apenas para dormir ou tomar banho. O dispositivo foi prendido a um cinto elástico e fixado no lado direito do quadril. Para análise, foi necessário um mínimo de 10 horas de registros de atividade física cotidiana. Foram considerados válidos aqueles que tivessem pelo menos 4 dias de atividade: 3 dias de semana e 1 dia de fim de semana. Os dados foram coletados na frequência de 30 Hz e analisados em épocas de 60 segundos. Períodos com valores consecutivos de 0 (com tolerância de pico de 2 min.) por 60 min. ou mais foram interpretados como “acelerômetro não usado” e excluídos da análise. A média do tempo total gasto em cada intensidade de atividade física foi calculada utilizando os pontos de corte específicos para idosos,^11^ adaptados por Buman et al.,^12^ considerando o tempo sedentário como 0 a 99 contagens/min; atividade física leve baixa como 100 a 1.040 contagens/min, atividade física leve alta como 1.041 a 1.951 contagens/min, e atividade física moderada a vigorosa como ≥ 1.952 contagens/min utilizando o eixo vertical, analisadas em min/dia, ajustando para a tempo e número de dias em que o dispositivo foi usado. Além disso, também calculamos a porcentagem de pacientes que atenderam às recomendações atuais de atividade física (≥ 150 min/semana) na linha de base e após 2 anos.

### Capacidade funcional

Foi realizado um teste de caminhada de 6 minutos em um corredor de 30 metros de comprimento, seguindo o protocolo descrito anteriormente.^13^ Dois cones foram colocados a 30 metros de distância e os pacientes foram orientados a caminhar o maior número possível de voltas ao redor dos cones. Também foram orientados a informar quando sintomas de claudicação (dor, desconforto, cãibras e cansaço) ocorreram para determinar a distância de início da claudicação. Além disso, a distância total percorrida foi definida como a distância máxima completada pelo paciente ao final do teste de caminhada de 6 minutos.

### Pressão arterial no consultório

A pressão arterial no consultório foi medida por meio de um monitor (HEM-742, Omron Healthcare, Japão), que consiste em um dispositivo eletrônico e digital de pressão arterial braquial com deflação e inflação automáticas. Para isso, os pacientes permaneceram sentados por pelo menos 10 minutos. Foram realizadas 3 medidas consecutivas, com intervalo de 1 minuto, em ambos os braços, com manguito de tamanho adequado. O valor utilizado foi a média das 3 medidas, conforme recomendado pela Sociedade Brasileira de Cardiologia.^14^

### Pressão arterial central

A pressão arterial central pela artéria radial por análise da onda de pulso utilizando a técnica de tonometria de aplanação (SphymoCor, AtCor Medical, Austrália). Após pelo menos 15 minutos de repouso em decúbito dorsal, foram utilizados 11 segundos de registro da onda de pressão arterial central radial. Após esse procedimento, o software SphygmoCor ® deriva a onda de pressão da aorta ascendente, equivalente à onda de pressão medida por um cateter invasivo, obtendo a pressão arterial central sistólica e diastólica. Para melhor acurácia da medida, foram considerados válidos apenas os valores com índices superiores a 90%.

### Rigidez arterial

A rigidez arterial foi estimada pela velocidade da onda de pulso carotídeo-femoral utilizando a técnica de tonometria de aplanação, seguindo as recomendações da *American Heart Association*.^15^ A velocidade da onda de pulso carotídeo-femoral foi registrada sequencialmente por transdutores transcutâneos posicionados acima da artéria carótida e da artéria femoral direita, utilizando um aparelho de tonometria de aplanação (Sphygmocor, AtCor Medical, Australia). O registro eletrocardiográfico foi obtido simultaneamente com as medidas das ondas de pulso carotídeo-femoral como padrão de referência para cálculo do tempo de trânsito da onda. Duas distâncias de superfície foram medidas pelo investigador: uma entre o ponto de registro na artéria carótida e uma na fúrcula esternal (distância 1) e outra entre a fúrcula esternal e a artéria femoral (distância 2). A distância percorrida pela onda de pulso foi calculada com a fórmula seguinte: distância 2 − distância 1. A velocidade da onda de pulso carotídeo-femoral foi calculada com a fórmula seguinte: velocidade da onda de pulso carotídeo-femoral = ¼ * distância percorrida pela onda de pulso (m) / tempo de trânsito (s).

### Modulação autonômica cardíaca

A modulação autonômica cardíaca foi avaliada pela técnica de variabilidade da frequência cardíaca. Para isso, os pacientes permaneceram em repouso, deitados por 15 minutos e os intervalos RR foram registrados por meio de um monitor de frequência cardíaca (Polar V800, Polar Electro, Finlândia). Para análise, os primeiros 5 minutos foram excluídos, e aqueles com pelo menos 10 minutos de sinal estável foram considerados sinais válidos. Após a coleta, os intervalos RR foram exportados para o programa Kubios HRV (Biosignal Analysis and Medical Imaging Group, Finlândia) e então analisados nos domínios de tempo e de frequência. Os parâmetros de domínio de tempo foram: desvio padrão de todos os intervalos (SDNN), raiz quadrada média das diferenças quadradas dos intervalos RR normais adjacentes (RMSSD) e porcentagem de intervalos adjacentes acima de 50 ms (PNN50).^16^ Os parâmetros de domínio de frequência foram obtidos pela técnica de análise espectral pelo método autorregressivo. As frequências entre 0,04 e 0,4 Hz foram consideradas fisiologicamente significativas; o componente de baixa frequência é representado por oscilações entre 0,04 e 0,15 Hz, e o componente de alta frequência por aquelas entre 0,15 e 0,4 Hz. A potência de cada componente espectral foi calculada em termos normalizados, dividindo-se a potência de cada banda pela potência total, da qual foi subtraído o valor da banda de frequência muito baixa (< 0,04 Hz), e o resultado foi multiplicado por 100.^16^

### Análise estatística

Todas as análises estatísticas foram realizadas usando o software Statistical Package for the Social Sciences e SPSS/PASW versão 20 (IBM Corp, New York, NY, EUA). Os dados de normalidade foram verificados pelo teste de Kolmogorov-Smirnov. As variáveis contínuas foram resumidas como média e desvio padrão (dados de distribuição normal) ou mediana e intervalo interquartil (dados de distribuição não normal), enquanto as variáveis categóricas foram resumidas como números absolutos e percentuais, com os respectivos intervalos de confiança.

Foram comparadas as características clínicas na linha de base e no acompanhamento usando o teste t pareado ou Wilcoxon *signed-rank* para variáveis contínuas e o teste de McNemar para variáveis categóricas. As associações entre as alterações delta (valores após 2 anos – valores da linha de base) na atividade física e nos parâmetros cardiovasculares foram analisadas por regressão linear múltipla ajustada para sexo, idade, mudanças na medicação anti-hipertensiva, ITB, peso e capacidade de caminhada, que são fatores de confusão clássicos na DAP.^17-20^

A análise residual foi realizada. A homocedasticidade foi analisada por análise gráfica (*scatterplot*), e a aderência à distribuição normal foi testada pelo teste de Kolmogorov-Smirnov. A análise de multicolinearidade foi realizada assumindo fatores de inflação de variância inferiores a 5 e tolerância inferior a 0,20. Para as análises, o nível de significância estatística foi estabelecida em p < 0,05.

## Resultados

**Figura 1:**
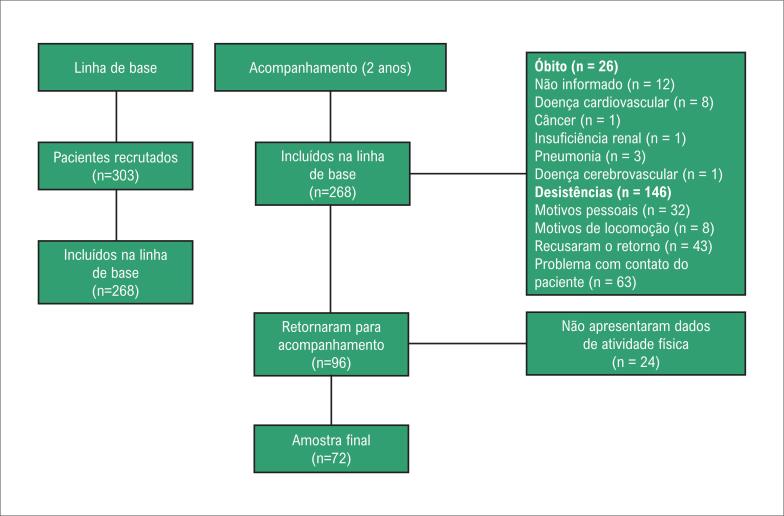
Fluxograma do estudo.

O recrutamento do estudo foi realizado entre setembro de 2015 e novembro de 2017 (Figura 1). Na primeira fase do estudo, 268 pacientes foram submetidos a medidas basais. Na segunda fase do estudo, 96 pacientes concordaram em participar e 24 destes pacientes não foram elegíveis por falta de dados sobre a atividade física. Portanto, a amostra final deste estudo é composta por 72 pacientes.

A Tabela 1 apresenta as características clínicas dos pacientes no início e no acompanhamento. Após 2 anos, observamos uma dimi-nuição do ITB em relacão aos valores de linha de base.

**Tabela 1:** Características clínicas de pacientes com doença arterial periférica (n = 72)

Variáveis	Linha de base	Acompanhamento	p
Peso (kg)	74,5±13,5	73,7±12,9	0,128
Índice de massa corporal (kg/m²)	27,5±4,4	27,3±4,0	0,357
Índice tornozelo-braquial	0,62±0,20	0,53±0,20	0,004
Teste de caminhada de 6 minutos	350±90	364±105	0,257
**Condições comórbidas**			
Diabetes mellitus (%)	42,3	47,2	0,375
Hipertensão (%)	82,9	86,1	0,500
Dislipidemia (%)	84,5	91,8	0,063
Obesidade (%)	26,3	38,0	0,359
Doença arterial coronária (%)	34,8	40,3	0,523
Acidente vascular encefálico (%)	15,7	21,9	0,125
Insuficiência cardíaca (%)	13,2	15,9	0,607
Câncer (%)	11,8	9,9	0,998
**Medicamento**			
Antiplaquetário (%)	89,7	84,5	0,549
Inibidor de ECA (%)	23,9	2,8	0,001
Antagonista do receptor de angiotensina (%)	27,9	28,2	0,727
Bloqueador dos canais de cálcio (%)	22,1	26,8	0,508
Diurético (%)	41,2	32,4	0,648
Betabloqueadores (%)	50,0	26,8	0,007
Estatinas (%)	92,6	90,1	0,774
Hipoglicêmicos (%)	47,1	42,3	0,727
Vasodilatador periférico (%)	29,4	47,9	0,004

Dados apresentados como média ± desvio padrão ou frequência relativa. ECA: enzima conversora de angiotensina.

A Tabela 2 apresenta os dados da atividade física na linha de base e no período de acompanhamento. Após 2 anos, observamos uma redução significativa no tempo gasto em atividade física total e aumento do tempo sedentário em relação aos valores de linha de base.

**Tabela 2:** Nível de atividade física dos pacientes na linha de base e no acompanhamento (n = 72)

Variáveis	Linha de base	Acompanhamento	p
Tempo sedentário	4178 (962)	4442 (809)	0,001
AF leve baixa (min/semana)	2055 (904)	1851 (662)	0,001
AF leve alta (min/semana)	2257,6 ± 774,5	2041 ± 676,2	0,001
AF moderada a vigorosa (min/semana)	85 (177)	41 (79)	0,001
AF total (min/semana)	2257,6 ± 774,5	2041 ± 676,2	0,001
Atendeu as recomendações de AF (n, %)	6 (7,8)	3 (3,9)	0,250

Dados apresentados como mediana (intervalo interquartil) ou como média ± desvio padrão. AF: atividade física.

A Tabela 3 apresenta os dados dos parâmetros de risco cardiovascular na linha de base e no acompanhamento. Observamos aumento da velocidade da onda de pulso carotídeo-femoral e diminuição do SDNN no acompanhamento quando comparados aos valores basais.

**Tabela 3:** Parâmetros de risco cardiovascular na linha de base e no acompanhamento (n = 72)

Variáveis	n	Linha de base	n	Acompanhamento	p
FC em repouso (bpm)	72	64,4± 11,5	72	67,7 ± 17,2	0,12
PAS braquial (mmHg)	72	133,3 ± 21,0	73	132,5 ± 21,0	0,69
PAD braquial (mmHg)	72	73,0 ± 10,2	73	72,7 ± 10,6	0,74
PA central (mmHg)	62	130,9 ± 22,3	62	128,0 ± 21,4	0,43
PAD central (mmHg)	62	75,2 ± 9,9	62	74,6 ± 9,8	0,79
PP (mmHg)	62	55,7 ± 182	62	52,5 ± 18,3	0,09
IA (%)	60	32,3 ± 11,1	60	30,6 ± 13,2	0,59
IA 75 bpm (%)	60	26,6 ± 9,6	60	26,9 ± 10,6	0,42
VOP-CF (m/s)	43	8,4 (3,21)	43	11,5 (6,2)	0,01
SDNN (ms)	39	45,6 ± 31,4	39	24,3 ± 13,3	0,01
RMSSD (ms)	39	31,7 (29,2)	39	21,1 (33,8)	0,18
PNN50 (%)	39	5,8 (16,8)	39	3,1 (18,5)	0,23
FB (un)	39	63,2 (32,4)	39	61,4 (24,6)	0,97
FA (un)	39	36,8 (32,4)	39	38,6 (24,6)	0,98
FB/FA	39	1,71 (3,11)	39	1,56 (1,69)	0,69

Dados apresentados como média ± desvio padrão ou como mediana (intervalo interquartil). FA: frequência alta; FB: frequência baixa; FC: frequência cardíaca; IA: índice de aumento; PA: pressão arterial; PAD: pressão arterial diastólica; PAS: pressão arterial sistólica; PNN50: porcentagem de intervalos adjacentes acima de 50 ms; PP: pressão de pulso; RMSSD: raiz quadrada média das diferenças quadradas dos intervalos RR normais adjacentes; SDNN: desvio padrão de todos os intervalos RR; VOP-CF: velocidade da onda de pulso carotídeo-femoral.

Não observamos associação entre tempo sedentário e atividade física com valores delta da pressão arterial clínica e da pressão arterial central (Tabela 3), indicadores de rigidez arterial e parâmetros de variabilidade da frequência cardíaca após 2 anos de acompanhamento em pacientes com DAP sintomática (Tabelas 4 e 5).

**Tabela 4:** Relação entre sedentarismo e atividade física com alterações na pressão arterial no consultório e central após 2 anos de acompanhamento em pacientes com doença arterial periférica sintomática (n = 72)

Variáveis independentes	Modelos	Δ PAS no consultório N=72		Δ PAD no consultório N=72		Δ PAS central N=62		Δ PAD central N=62	
		b	p	b	p	b	p	b	p
Δ Tempo sedentário (min/semana)	Bruto	0,045	0,707	-0,079	0,512	0,085	0,518	0,113	0,391
	Ajustado	0,172	0,254	-0,117	0,907	0,235	0,183	0,211	0,230
Δ AF leve baixa (min/semana)	Bruto	-0,075	0,531	0,055	0,646	-0,109	0,407	-0,106	0,419
	Ajustado	-0,193	0,202	0,010	0,947	-0,256	0,146	-0,196	0,275
Δ AF leve alta (min/semana)	Bruto	-0,001	0,933	-0,005	0,274	0,001	0,906	0,002	0,726
	Ajustado	-0,002	0,895	-0,005	0,357	-0,003	0,843	0,002	0,784
Δ AFMV (min/semana)	Bruto	0,054	0,653	0,039	0,746	-0,042	0,749	-0,122	0,352
	Ajustado	0,250	0,098	0,227	0,120	0,194	0,270	-0,044	0,806

Todas as análises foram ajustadas para sexo, idade, mudanças na medicação anti-hipertensiva, índice tornozelo-braquial, peso e capacidade de caminhada. AF: atividade física; AFMV: atividade física moderada a vigorosa; b: coeficientes padronizados; PAD: pressão arterial diastólica; PAS: pressão arterial sistólica.

**Tabela 5:** Relação entre sedentarismo e atividade física com alterações nos indicadores de rigidez arterial e nos parâmetros de variabilidade da frequência cardíaca após 2 anos de acompanhamento em pacientes com doença arterial periférica sintomática (n = 72)

Variáveis independentes	Modelos	Δ VOP-CF N=43		Δ IA N=60		Δ SDNN N=39		Δ FB/FA N=39		Δ FB N=39		Δ FA N=39	
		b	p	b	p	b	p	b	p	b	p	b	p
Δ Tempo sedentário (min/semana)	Bruto	-0,148	0,349	0,129	0,331	-0,004	0,557	0,087	0,608	0,001	0,923	-0,001	0,923
	Ajustado	-0,003	0,989	0,100	0,568	-0,007	0,458	-0,061	0,842	-0,002	0,841	0,002	0,841
Δ AF leve baixa (min/semana)	Bruto	0,154	0,330	-0,168	0,203	-0,003	0,596	-0,081	0,634	-0,001	0,837	0,001	0,837
	Ajustado	-0,018	0,936	-0,188	0,279	0,010	0,416	0,066	0,829	0,001	0,911	-0,001	0,911
Δ AF leve alta (min/semana)	Bruto	0,001	0,002	-0,008	0,245	-0,023	0,179	-0,001	0,506	-0,003	0,814	0,003	0,814
	Ajustado	0,002	0,477	-0,006	0,443	-0,019	0,359	-0,002	0,444	-0,021	0,286	0,021	0,286
Δ AFMV (min/semana)	Bruto	-0,070	0,660	0,150	0,256	0,007	0,901	-0,240	0,153	-0,042	0,352	0,042	0,352
	Ajustado	-0,028	0,897	0,038	0,194	-0,019	0,773	-0,196	0,415	-0,015	0,814	0,015	0,814

Todas as análises foram ajustadas para sexo, idade, mudanças na medicação anti-hipertensiva, índice tornozelo-braquial, peso e capacidade de caminhada. AF: atividade física; AFMV: atividade física moderada a vigorosa; b: coeficientes padronizados; FA: frequência alta; FB: frequência baixa; IA: índice de aumento; VOP-CF: velocidade da onda de pulso carotídeo-femoral; SDNN: desvio padrão de todos os intervalos RR.

## Discussão

Os resultados do presente estudo indicam que ocorrem alterações importantes nos parâmetros de risco cardiovascular e na atividade física após 2 anos em pacientes com DAP sintomática. Tais alterações incluem aumento da prevalência de comorbidades, diminuição hemodinâmica dos membros inferiores (ITB), aumento da rigidez arterial e redução dos níveis de atividade física com concomitante aumento do tempo gasto em comportamento sedentário.

Os resultados também indicam uma acentuada piora do perfil clínico na nossa amostra, com aumento da prevalência de fatores de risco cardiovascular após 2 anos de acompanhamento. Também foram observadas redução do ITB e variabilidade da frequência cardíaca, bem como aumento da rigidez arterial. Como esses fatores estão altamente relacionados à mortalidade cardiovascular,^21-23^ as alterações no perfil clínico e nos parâmetros cardiovasculares observadas ao longo do tempo em pacientes com DAP podem explicar potencialmente o prognóstico grave desses pacientes. Dessa maneira, esses resultados destacam a importância de estratégias agressivas de prevenção secundária, incluindo modificação de fatores de risco, terapia antiplaquetária, terapia hipolipemiante, tratamento anti-hipertensivo e, principalmente, aumento nos níveis de atividade física.^24,25^ De fato, estudos prévios mostraram que atividade física regular melhorou diversos parâmetros de saúde em pacientes com DAP, tais como capacidade de caminhar, função vascular, inflamação e saturação de oxigênio da hemoglobina do músculo da panturrilha.^26-28^

As diretrizes de atividade física para a população geral e com DAP recomendam praticar pelo menos 150 minutos de atividade física moderada, 75 minutos de atividade física vigorosa ou uma combinação equivalente de atividade física moderada a vigorosa semanalmente para promover benefícios gerais à saúde.^24-26^ No presente estudo, durante o acompanhamento de 2 anos, os pacientes aumentaram o tempo sedentário em 7%, enquanto a atividade física leve baixa, leve alta, moderada a vigorosa e total, diminuíram 7%, 10%, 38% e 10%, respectivamente. Além disso, foi observada redução de 50% dos pacientes que atenderam às recomendações das diretrizes de atividade física após 2 anos de acompanhamento (7,8% versus 3,9%). Esses resultados são alarmantes, tendo em vista que as diretrizes para pacientes com DAP são claras ao recomendar a atividade física regular como um tratamento clínico inicial.^29,30^ Dessa maneira, como a maioria de nossos pacientes não modificou ou mesmo piorou seus níveis de atividade física, surge a necessidade de explorar estratégias para compreender as possíveis barreiras e criar novas estratégias para promover a prática de atividade física nesses pacientes.

Não observamos associação entre as alterações na atividade física com nenhum dos parâmetros cardiovasculares durante o acompanhamento de 2 anos. Esses resultados contrastam com a nossa hipótese inicial de que as alterações na atividade física estariam associadas aos parâmetros de risco cardiovascular. Uma possível explicação é que a maioria de nossos pacientes já estava fisicamente inativa na linha de base e apenas 3,9% atenderam às recomendações mínimas de atividade física durante o acompanhamento. Assim, esses níveis mais baixos de atividade física não foram suficientes para promover alterações nos parâmetros de risco cardiovascular em pacientes com DAP durante o período de acompanhamento.

Este estudo é uma análise de um acompanhamento de 2 anos. Os resultados são preliminares e requerem investigações adicionais em um período de acompanhamento maior e em uma amostra maior. O significado clínico do presente estudo é que esses pacientes apresentaram perfil cardiovascular comprometido e atividade física reduzida após 2 anos e esses resultados destacam a importância de desenvolver e implementar estratégias para enfrentar esses fatores de risco com o objetivo de reduzir o risco cardiovascular na população com DAP.

Este estudo apresenta algumas limitações que devem ser mencionadas. Tivemos uma perda significativa de dados de variabilidade da frequência cardíaca devido à presença de arritmias cardíacas ou marca-passos, o que pode ter afetado o poder de inferir causa e efeito para essas variáveis. Em alguns pacientes, não foi possível coletar os dados da tonometria de aplanação devido ao pulso femoral não detectável (pulso fraco ou inexistente). Tivemos altas taxas de desistência durante o período de acompanhamento, o que pode incorrer em um viés de seleção. Por outro lado, pontos fortes do nosso estudo incluem o desenho longitudinal de 2 anos, análise mais robusta dos parâmetros de risco cardiovascular e a medição objetiva dos níveis de atividade física.

## Conclusão

Os pacientes com DAP apresentaram níveis reduzidos de atividade física e comprometimento em relação aos parâmetros de risco cardiovascular após 2 anos. Além disso, não houve associação de alterações na atividade física com os parâmetros de risco cardiovascular ao longo de 2 anos de acompanhamento.
