# Supplementary material for: Alterações Longitudinais nos Níveis de Atividade Física e Parâmetros de Risco Cardiovascular em Pacientes com Doença Arterial Periférica Sintomática
Source: Arq Bras Cardiol. 2022 May 24;119(1):59–66. [Article in Portuguese] doi: 10.36660/abc.20210386 (PMC9352116; doi:10.36660/abc.20210386)
Supplement: Translation

# Translation: Longitudinal Changes in Physical Activity Levels and Cardiovascular Risk Parameters in Patients with Symptomatic Peripheral Artery Disease

**Authors:** Francielly Monteiro, Marilia de Almeida Correia, Breno Quintella Farah, Diego Giuliano Destro Christofaro, Paulo Mesquita Longano de Oliveira, Raphael Mendes Ritti-Dias, Gabriel Grizzo Cucato

**Affiliations:** 1 São Paulo SP Brazil Hospital Israelita Albert Einstein - Instituto de Educação e Pesquisa, São Paulo, SP – Brazil; 2 São Paulo SP Brazil Universidade Nove de Julho - Programa de Pós-graduação em Medicina, São Paulo, SP – Brazil; 3 Recife PE Brazil Universidade Federal Rural de Pernambuco, Recife, PE – Brazil; 4 Presidente Prudente SP Brazil Universidade Estadual Paulista Júlio de Mesquita Filho, Presidente Prudente, SP – Brazil; 5 Newcastle upon Tyne United Kingdom Northumbria University, Newcastle upon Tyne – United Kingdom

**Keywords:** Peripheral Arterial Disease, Cardiovascular System, Arterial Pressure, Exercise

## Abstract

**Background:**

Previous cross-sectional studies have demonstrated that physical activity is associated with lower cardiovascular risk in patients with peripheral artery disease (PAD). However, it is not possible to establish causality, and longitudinal design studies are required.

**Objective:**

To analyze the changes in cardiovascular risk parameters and physical activity levels after a 2-year follow-up in patients with symptomatic PAD.

**Methods:**

This study started in 2015. In the first phase, 268 patients were included. In the second phase, after 2 years (median = 26 months), 72 patients were re-evaluated. Cardiovascular risk parameters, such as blood pressure, cardiac autonomic modulation, and arterial stiffness, and physical activity levels were measured at baseline and after 2 years of follow-up. Association among delta changes (values from follow-up – baseline) in physical activity and cardiovascular parameters were analyzed by multiple linear regression. The significance level was set at p < 0.05.

**Results:**

Patients reduced their total physical activity levels compared to baseline (baseline = 2257.6 ± 774.5 versus follow-up = 2041 ± 676.2 min/week, p = 0.001). After follow-up, ankle-brachial index (0.62 ± 0.20 versus 0.54 ± 0.20, p = 0.003), and standard deviation of all RR intervals (43.4 ± 27.0 versus 25.1 ± 13.4 ms, p < 0.001) were lower, whereas carotid-femoral pulse wave velocity was higher (9.0 ± 3.0 versus 10.7 ± 3.4 m/s, p = 0.002) compared to baseline values. We did not observe any association among delta values of physical activity levels and cardiovascular risk parameters.

**Conclusion:**

Patients with PAD had reduced physical activity levels and impaired cardiovascular risk parameters during 2-year follow-up.

## Introduction

Intermittent claudication is the main symptom of peripheral artery disease (PAD), and it is characterized by pain, cramps, or a burning sensation that affects lower limbs during physical activity, especially while walking.^1^ Patients with PAD and intermittent claudication symptoms present limited mobility, poor control of cardiovascular parameters,^2,3^ and impaired quality of life.^4,5^

Physical activity has been recommended to improve functional capacity and cardiovascular function in these patients.^6-8^ In fact, patients with symptomatic PAD and higher levels of physical activity present better functional capacity and a lower risk of cardiovascular mortality compared to sedentary patients.^9,10^ However, due to the cross-sectional design of these studies, it is not possible to establish causality, and longitudinal design studies are required. Also, it is unknown whether alterations in these parameters occur during follow-up in PAD patients.

Therefore, this study aimed to analyze the longitudinal changes in physical activity and cardiovascular risk parameters after a 2-year follow-up in patients with PAD. We also analyzed whether changes in physical activity levels are associated with changes in cardiovascular risk parameters after a 2-year follow-up. We hypothesized that changes in physical activity levels would be associated with better cardiovascular risk parameters.

## Methods

This is a longitudinal study that started in 2015, consisting of 2 phases. In the first phase of the study, 268 patients were included and submitted to measurements of physical activity (accelerometry), functional capacity, and cardiovascular risk parameters (clinical blood pressure, central blood pressure, cardiac autonomic modulation, and arterial stiffness). After 2 years, all patients included in the first phase were invited to phase 2.

### Sample recruitment, screening, and sizing

Patients were recruited at hospitals in Sao Paulo, Brazil. The inclusion criteria were: age > 45 years of both sexes, ankle-brachial index (ABI) < 0.90 in one or both limbs, and presence of intermittent claudication symptoms. This study was approved by the Institutional Ethics Committee. Before data collection, patients were informed about the procedures involved in the study, and they signed an informed consent form.

Before and after the 2-year follow-up, patients underwent evaluations in 2 visits with an interval of at least 7 days. During the first visit, clinical, socio-demographic, and functional capacity data were obtained, and all patients received a physical activity monitor GT3X+ triaxial accelerometer (Actigraph, Pensacola, FL, USA). During the second visit, measurements of cardiovascular risk parameters such as clinical blood pressure, central blood pressure, cardiac autonomic modulation, and arterial stiffness were obtained. This session started between 1:00 and 2:00 pm, and patients were given the following instructions: eat a light meal, do not exercise at least 24 hours before the day of the evaluation, do not drink any alcoholic or caffeinated drinks, do not smoke 12 hours before the session, and maintain a normal routine of eating and taking their medication.

### Physical activity level

Physical activity levels were obtained using a GT3X+ triaxial accelerometer (Actigraph, Pensacola, FL, USA). All patients received instructions to use the accelerometer for 7 consecutive days, removing it only for sleeping or bathing. The device was attached to an elastic belt and fixed to the right side of the hip. For analysis, a minimum of 10 hours of daily physical activity recordings was necessary. They were considered valid if they had at least 4 days of activity: 3 weekdays and 1 weekend day. The data were collected in the frequency of 30 Hz and were analyzed using 60-second epochs. Periods with consecutive values of 0 (with a 2 min spike tolerance) for 60 min or longer were interpreted as “accelerometer not worn” and excluded from the analysis. The average of total time spent at each intensity of physical activity was calculated using the cutoff points specific to older people,^11^ adapted by Buman et al.,^12^ considering sedentary time as 0 to 99 counts/min; low-light physical activity as 100 to 1040 counts/min, high-light physical activity as 1041 to 1951 counts/min, and moderate to vigorous physical activity as ≥ 1952 counts/min using the vertical axis, analyzed in min/day, adjusting for the time and number of days the device was worn. Additionally, we also calculated the percentage of patients who met the current physical activity recommendations (≥ 150 min/week) at baseline and after 2 years.

### Functional capacity

A 6-minute walk test was conducted in a 30-meter long corridor, following the protocol previously described.^13^ Two cones were placed 30 meters apart, and patients were instructed to walk as many laps around the cones as possible. They were also instructed to inform when claudication symptoms (pain, discomfort, cramps, and tiredness) occurred in order to determine claudication onset distance. In addition, the total walking distance was defined as the maximum distance completed by the patient at the end of the 6-minute walk test.

### Office blood pressure

Office blood pressure was measured using a monitor (HEM-742, Omron Healthcare, Japan), which consists of an electronic and digital arm blood pressure device with automatic deflation and inflation. For this, patients remained in a sitting position for at least 10 minutes. Three consecutive measurements were taken, 1 minute apart, on both arms, with adequate cuff size. The value used was the average of the 3 measurements, as recommended by the Brazilian Society of Cardiology.^14^

### Central blood pressure

Central blood pressure was measured by radial artery by pulse wave analysis using the applanation tonometry technique (SphymoCor, AtCor Medical, Australia). After at least 15 minutes of rest in the supine position, 11 seconds of radial central blood pressure wave recording were used. After this procedure, the SphygmoCor ® software derives the ascending aorta pressure wave, equivalent to the pressure wave measured by an invasive catheter, obtaining systolic and diastolic central blood pressure. For better measurement accuracy, only values with indexes greater than 90% were considered valid.

### Arterial stiffness

Arterial stiffness was estimated by carotid-femoral aortic pulse wave velocity using the applanation tonometry technique, following the recommendations of the American Heart Association.^15^ The carotid-femoral aortic pulse wave velocity was recorded sequentially by transcutaneous transducers positioned above the carotid artery and the right femoral artery, using an applanation tonometry apparatus (Sphygmocor, AtCor Medical, Australia). Electrocardiography recording was obtained simultaneously with carotid-femoral aortic pulse wave measurements as a reference standard for calculating wave transit time. Two surface distances were measured by the investigator: one between the recording point in the carotid artery and the sternal notch (distance 1) and the other between the sternal notch and femoral artery (distance 2). The distance travelled by the pulse wave was calculated as: distance 2 − distance 1. Carotid-femoral aortic pulse wave velocity was calculated as: carotid-femoral aortic pulse wave velocity = ¼ * distance travelled by pulse wave (m) / transit time (s).

### Cardiac autonomic modulation

Cardiac autonomic modulation was assessed by the heart rate variability technique. For this, patients remained at rest, lying down for 15 minutes and the RR intervals were recorded using a heart rate monitor (Polar V800, Polar Electro, Finland). For analysis, the first 5 minutes were excluded, and those with at least 10 minutes of steady signal were considered valid signals. After collection, RR intervals were exported to Kubios HRV program (Biosignal Analysis and Medical Imaging Group, Finland) and then analyzed in time and frequency domains. Time-domain parameters were: standard deviation of all RR intervals (SDNN), root mean square of the squared differences between adjacent normal RR intervals (RMSSD), and percentage of adjacent intervals over 50 ms (PNN50).^16^ Frequency domain parameters were obtained by the spectral analysis technique using the autoregressive method. Frequencies between 0.04 and 0.4 Hz were considered physiologically significant; the low-frequency component is represented by oscillations between 0.04 and 0.15 Hz, and the high-frequency component by those between 0.15 and 0.4 Hz. The power of each spectral component was calculated in normalized terms, dividing the power of each band by the total power, from which the very low frequency (< 0.04 Hz) band value was subtracted, and the result was multiplied by 100.^16^

### Statistical analysis

All statistical analyses were performed using Statistical Package for the Social Sciences software and SPSS/PASW version 20 (IBM Corp, New York, NY, USA). Normality data were analyzed using the Kolmogorov-Smirnov test. Continuous variables were summarized as mean and standard deviation (normal distribution data) or median and interquartile range (non-normal distribution data), whereas categorical variables were summarized as absolute numbers and percentages, with the respective confidence intervals.

Clinical characteristics at baseline and follow-up were compared using the paired t-test or Wilcoxon signed-rank for continuous variables and the McNemar test for categorical variables. Associations among the delta changes (values from follow-up – baseline) in physical activity and cardiovascular parameters were analyzed by multiple linear regression adjusted for sex, age, changes in antihypertensive medication, ABI, weight, and walking capacity, which are classical confounders in PAD.^17-20^

Residual analysis was performed. Homoscedasticity was analyzed by graphical analysis (scatterplot), and adherence to normal distribution was tested using the Kolmogorov-Smirnov test. Multicollinearity analysis was performed assuming variance inflation factors less than 5 and tolerance below 0.20. For analyses, significance level was set at p < 0.05.

## Results

The recruitment of the study was conducted between September 2015 and November 2017 (Figure 1). In the first phase of the study, 268 patients underwent baseline measurements. In the second phase of the study, 96 patients agreed to participate, and 24 of those patients were not eligible because of missing data on physical activity. Therefore, the final sample of this study comprises 72 patients.

**Figure 1:** Flowchart of the study.

Table 1 shows the clinical characteristics of patients at baseline and follow-up. After 2 years, we observed a decrease in ABI during follow-up.

**Table 1:** Clinical characteristics of patients with peripheral artery disease (n = 72)

Variables	Baseline	Follow-up	p
Weight (kg)	74.5±13.5	73.7±12.9	0.128
Body mass index (kg/m²)	27.5±4.4	27.3±4.0	0.357
Ankle-brachial index	0.62±0.20	0.53±0.20	0.004
Six-minute walk test	350±90	364±105	0.257
**Comorbid conditions**			
Diabetes mellitus (%)	42.3	47.2	0.375
Hypertension (%)	82.9	86.1	0.500
Dyslipidemia (%)	84.5	91.8	0.063
Obesity (%)	26.3	38.0	0.359
Coronary artery disease (%)	34.8	40.3	0.523
Stroke (%)	15.7	21.9	0.125
Heart failure (%)	13.2	15.9	0.607
Cancer (%)	11.8	9.9	0.998
**Medication**			
Antiplatelet (%)	89.7	84.5	0.549
ACE inhibitor (%)	23.9	2.8	0.001
Angiotensin-receptor antagonist (%)	27.9	28.2	0.727
Calcium-channel blocker (%)	22.1	26.8	0.508
Diuretic (%)	41.2	32.4	0.648
Beta-blockers (%)	50.0	26.8	0.007
Statins (%)	92.6	90.1	0.774
Hypoglycemics (%)	47.1	42.3	0.727
Peripheral vasodilator (%)	29.4	47.9	0.004

Data presented as mean ± standard deviation or relative frequency. ACE: angiotensin-converting enzyme.

Table 2 shows data on total physical activity at the baseline and follow-up period. After 2 years, we observed a significant reduction in time spent in total physical activity and an increase in sedentary time compared to baseline values.

**Table 2:** Physical activity level of patients at baseline and follow-up (n = 72)

Variables	Baseline	Follow-up	p
Sedentary time	4178 (962)	4442 (809)	0.001
Low-light PA (min/week)	2055 (904)	1851 (662)	0.001
High-light PA (min/week)	2257.6 ± 774.5	2041 ± 676.2	0.001
Moderate to vigorous PA (min/week)	85 (177)	41 (79)	0.001
Total PA (min/week)	2257.6 ± 774.5	2041 ± 676.2	0.001
Met PA recommendations (n, %)	6 (7.8)	3 (3.9)	0.250

Data presented as median (interquartile range) or as mean ± standard deviation. PA: physical activity.

Table 3 shows data on cardiovascular risk parameters at baseline and follow-up. We observed an increase in carotid-femoral pulse wave velocity and a decrease in SDNN in follow-up when compared to baseline values.

**Table 3:** Cardiovascular risk parameters at baseline and follow-up (n = 72)

Variables	n	Baseline	n	Follow-up	p
Resting HR (bpm)	72	64.4± 11.5	72	67.7 ± 17.2	0.12
Brachial SBP (mmHg)	72	133.3 ± 21.0	73	132.5 ± 21.0	0.69
Brachial DBP (mmHg)	72	73.0 ± 10.2	73	72.7 ± 10.6	0.74
Central BP (mmHg)	62	130.9 ± 22.3	62	128.0 ± 21.4	0.43
Central DBP (mmHg)	62	75.2 ± 9.9	62	74.6 ± 9.8	0.79
PP (mmHg)	62	55.7 ± 182	62	52.5 ± 18.3	0.09
AIx (%)	60	32.3 ± 11.1	60	30.6 ± 13.2	0.59
AIx 75 bpm (%)	60	26.6 ± 9.6	60	26.9 ± 10.6	0.42
Cf-PWV (m/s)	43	8.4 (3.21)	43	11.5 (6.2)	0.01
SDNN (ms)	39	45.6 ± 31.4	39	24.3 ± 13.3	0.01
RMSSD (ms)	39	31.7 (29.2)	39	21.1 (33.8)	0.18
PNN50 (%)	39	5.8 (16.8)	39	3.1 (18.5)	0.23
LF (un)	39	63.2 (32.4)	39	61.4 (24.6)	0.97
HF (un)	39	36.8 (32.4)	39	38.6 (24.6)	0.98
LF/HF	39	1.71 (3.11)	39	1.56 (1.69)	0.69

Data presented as mean ± standard deviation or as median (interquartile range). AIx: augmentation index; BP: blood pressure; Cf-PWV: carotid-femoral pulse wave velocity; DBP: diastolic blood pressure; HF: high frequency; HR: heart rate; LF: low frequency; PNN50: percentage of adjacent intervals over 50 ms; PP: pulse pressure; RMSSD: root mean square of the squared differences between adjacent normal RR intervals; SBP: systolic blood pressure; SDNN: standard deviation of all RR intervals.

We did not observe any association between sedentary time and physical activity with delta values of office and central blood pressure (Table 3), arterial stiffness indicators, and heart rate variability parameters after 2-year follow-up in patients with symptomatic PAD (Tables 4 and 5).

**Table 4:** Relationship between sedentary and physical activity with changes in office and central blood pressure after 2-year follow-up in patients with symptomatic peripheral artery disease (n = 72)

Independent variables	Models	Δ Office SBP N=72		Δ Office DBP N=72		Δ Central SBP N=62		Δ Central DBP N=62	
		b	p	b	p	b	p	b	p
Δ Sedentary time (min/week)	Crude	0.045	0.707	-0.079	0.512	0.085	0.518	0.113	0.391
	Adjusted	0.172	0.254	-0.117	0.907	0.235	0.183	0.211	0.230
Δ Low-light PA (min/week)	Crude	-0.075	0.531	0.055	0.646	-0.109	0.407	-0.106	0.419
	Adjusted	-0.193	0.202	0.010	0.947	-0.256	0.146	-0.196	0.275
Δ High-light PA (min/week)	Crude	-0.001	0.933	-0.005	0.274	0.001	0.906	0.002	0.726
	Adjusted	-0.002	0.895	-0.005	0.357	-0.003	0.843	0.002	0.784
Δ MVPA (min/week)	Crude	0.054	0.653	0.039	0.746	-0.042	0.749	-0.122	0.352
	Adjusted	0.250	0.098	0.227	0.120	0.194	0.270	-0.044	0.806

All analyses were adjusted for sex, age, changes in antihypertensive medication, ankle-brachial index, weight, and walking capacity. b: standardized coefficients; DBP: diastolic blood pressure; MVPA: moderate to vigorous physical activity; PA: physical activity; SBP: systolic blood pressure.

**Table 5:** Relationship between sedentary and physical activity with changes arterial stiffness indicators and heart rate variability parameters after 2-year follow-up in patients with symptomatic peripheral artery disease (n = 72)

Independent variables	Models	Δ Cf-PWV N=43		Δ AIx N=60		Δ SDNN N=39		Δ LF/HF N=39		Δ LF N=39		Δ HF N=39	
		b	p	b	p	b	p	b	p	b	p	b	p
Δ Sedentary time (min/week)	Crude	-0.148	0.349	0.129	0.331	-0.004	0.557	0.087	0.608	0.001	0.923	-0.001	0.923
	Adjusted	-0.003	0.989	0.100	0.568	-0.007	0.458	-0.061	0.842	-0.002	0.841	0.002	0.841
Δ Low-light PA (min/week)	Crude	0.154	0.330	-0.168	0.203	-0.003	0.596	-0.081	0.634	-0.001	0.837	0.001	0.837
	Adjusted	-0.018	0.936	-0.188	0.279	0.010	0.416	0.066	0.829	0.001	0.911	-0.001	0.911
Δ High-light PA (min/week)	Crude	0.001	0.002	-0.008	0.245	-0.023	0.179	-0.001	0.506	-0.003	0.814	0.003	0.814
	Adjusted	0.002	0.477	-0.006	0.443	-0.019	0.359	-0.002	0.444	-0.021	0.286	0.021	0.286
Δ MVPA (min/week)	Crude	-0.070	0.660	0.150	0.256	0.007	0.901	-0.240	0.153	-0.042	0.352	0.042	0.352
	Adjusted	-0.028	0.897	0.038	0.194	-0.019	0.773	-0.196	0.415	-0.015	0.814	0.015	0.814

All analyses were adjusted for sex, age, changes in antihypertensive medication, ankle-brachial index, weight, and walking capacity. AIx: augmentation index; b: standardized coefficients; Cf-PWV: carotid-femoral pulse wave velocity; HF: high frequency; LF: low frequency; MVPA: moderate to vigorous physical activity; PA: physical activity; SDNN: standard deviation of all RR intervals.

## Discussion

The results of this study indicate that important changes in cardiovascular risk parameters and physical activity occurs after 2 years in patients with symptomatic PAD. These changes include increases in the prevalence of comorbid conditions, decreases in lower limb hemodynamic (ABI), increases in arterial stiffness, and reductions in physical activity levels with a concomitant increase in time spent in sedentary behavior.

The results also indicate a marked worsening in the clinical profile in our sample, with an increase in the prevalence of cardiovascular risk factors after a 2-year follow-up. Reduced ABI and heart rate variability and increased arterial stiffness were also observed. As these factors are highly related to cardiovascular mortality,^21-23^ the alterations in clinical profile and cardiovascular parameters observed over time in patients with PAD may potentially explain the severe prognosis of these patients. Thus, these results highlight the importance of aggressive secondary prevention strategies, including risk factor modification, antiplatelet therapy, lipid-lowering therapy, antihypertensive treatment, and especially increased physical activity levels.^24,25^ In fact, previous studies have shown that regular physical activity improved different health parameters in PAD, such as walking ability, vascular function, inflammation, and calf muscle hemoglobin oxygen saturation.^26-28^

Physical activity guidelines for the general and PAD population recommend engaging in at least 150 minutes of moderate physical activity, 75 minutes of vigorous physical activity, or an equivalent combination of moderate to vigorous physical activity weekly to promote overall health benefits.^24-26^ In the present study, during the 2-year follow-up, patients increased their sedentary time 7% while in low-light, high-light, moderate to vigorous, and total physical activity, they decreased 7%, 10%, 38%, and 10%, respectively. In addition, a reduction of 50% of patients who met the recommendations for physical activity guidelines was observed after a 2-year follow-up (7.8% versus 3.9%). These results are alarming since the guidelines for patients with PAD are clear in recommending regular physical activity as an initial clinical treatment.^29,30^ Thus, as most of our patients did not modify or even worsened their physical activity levels, this raises the need to explore strategies to understand the barrier and create new strategies to promote engagement in physical activity in these patients.

We did not observe an association between changes in physical activity with any of the cardiovascular parameters during the 2-year follow-up. These results contrast with our initial hypothesis that changes in physical activity would be associated with cardiovascular risk parameters. A possible explanation is that most of our patients were already physically inactive at baseline, and only 3.9% met the minimum physical activity recommendations during the follow-up. Thus, these lower levels of physical activity were not enough to promote changes in cardiovascular risk parameters in patients with PAD during the follow-up period.

This study is an analysis of a 2-year follow-up, and the results are preliminary and require further investigations at a longer follow-up period and in a larger sample size. The clinical significance of the present study is that these patients presented impaired cardiovascular profile and reduced physical activity after 2 years, and these results highlight the importance of developing and delivering clinical strategies to tackle these risk factors with the aim of reducing cardiovascular risk in the PAD population.

This study has some limitations that should be mentioned. We had a significant loss of heart rate variability data due to the presence of cardiac arrhythmias or pacemakers, which may have affected the power to infer cause and effect for these variables. In some patients, it was not possible to collect the applanation tonometry data because of a non-detectable femoral pulse (weak or nonexistent pulse). We had high dropout rates during the follow-up period, which may incur a selection bias. On the other hand, strong aspects of our study include the 2-year longitudinal design, more robust analysis of cardiovascular risk parameters, and the objective measurement of physical activity levels.

## Conclusion

Patients with PAD had reduced physical activity levels and impaired cardiovascular risk parameters after 2 years. In addition, there was no association of changes in physical activity with cardiovascular risk parameters over the 2-year follow-up.
